# CRISPR/Cas12a-Based Biosensing: Advances in Mechanisms and Applications for Nucleic Acid Detection

**DOI:** 10.3390/bios15060360

**Published:** 2025-06-04

**Authors:** Kun Du, Qinlong Zeng, Mingjun Jiang, Zhiqing Hu, Miaojin Zhou, Kun Xia

**Affiliations:** 1 MOE Key Laboratory of Rare Pediatric Diseases, College of Basic Medical Sciences, Hengyang Medical School, University of South China, Hengyang 421009, China; 20222131112157@stu.usc.edu.cn (K.D.); huzhiqing@usc.edu.cn (Z.H.); 2 Department of Psychiatry, The Seventh Affiliated Hospital, Hengyang Medical School, University of South China (Hunan Provincial Veterans Administration Hospital), Changsha 410006, China; 3 Innovation Center for Diagnostics and Treatment of Thalassemia, Nanfang Hospital, Southern Medical University, Guangzhou 510515, China; zengqinlong@sklmg.edu.cn; 4 The Second Affiliated Hospital, Department of Vascular Surgery, Hengyang Medical School, University of South China, Hengyang 421099, China; 20222102111714@stu.usc.edu.cn; 5 Center for Medical Genetics and Hunan Key Laboratory of Medical Genetics, School of Life Sciences, Central South University, Changsha 410078, China

**Keywords:** CRISPR/Cas12a-based biosensors, nucleic acid detection, universal screening, isothermal amplification

## Abstract

Nucleic acid detection technology is crucial for molecular diagnosis. The advent of CRISPR/Cas12a-based nucleic acid detection has considerably broadened its scope, from the identification of infectious disease-causing microorganisms to the detection of disease-associated biomarkers. This innovative system capitalizes on the non-specific single-strand cleavage activity of Cas12a upon target DNA recognition. By employing a fluorescent probe in the form of a single-stranded DNA/RNA, this technology enables the observation of fluorescence changes resulting from nonspecific cleavage, thereby facilitating detection. CRISPR/Cas12a-based detection systems can be regarded as a new type of biosensor, offering a practical and efficient approach for nucleic acid analysis in various diagnostic settings. CRISPR/Cas12a-based biosensors outperform conventional nucleic acid detection methods in terms of portability, simplicity, speed, and efficiency. In this review, we elucidate the detection principle of CRISPR/Cas12a-based biosensors and their application in disease diagnostics and discuss recent innovations and technological challenges, aiming to provide insights for the research and further development of CRISPR/Cas12a-based biosensors in personalized medicine. Our findings show that although CRISPR/Cas12a-based biosensors have considerable potential for various applications and theoretical research, certain challenges remain. These include simplifying the reaction process, enhancing precision, broadening the scope of disease detection, and facilitating the translation of research findings into clinical practice. We anticipate that ongoing advancements in CRISPR/Cas12a-based biosensors will address these challenges.

## 1. Introduction

Nucleic acid detection technologies have been widely used in food safety testing, environmental surveillance, clinical diagnostics, and agricultural technology and have marked substantial advancements in molecular biology [1,2,3,4]. The scope of clinical applications has expanded beyond microbial and parasitic detection to the detection of disease-specific biomarkers across various biological systems [5,6,7]. Numerous techniques have been developed for nucleic acid detection, including PCR-based methods (qPCR, dPCR) [8,9,10,11,12], nucleic acid hybridization-based approaches (ISH, FISH, MLPA) [13,14], and nucleic acid sequencing-based procedures (Sanger, NGS) [15]. However, although the technical principles and operation methods differ, all these technical platforms rely on bulky instruments and well-trained experimenters and are time-consuming, which limits their utility in widespread screening, particularly in remote or under-resourced areas.

Clustered regularly interspaced short palindromic repeats (CRISPR) and CRISPR-associated (Cas) proteins facilitate adaptive immunity in bacteria and archaea [16,17] and have emerged as versatile tools in biotechnology, with applications extending to gene editing and nucleic acid detection. The system is divided into two primary classes—class 1, which requires multiple effector proteins for targeted DNA cleavage and is involved in a singular aspect of the immune defense, including types I, III, IV, and III; class 2, which employs a single RNA-guided endonuclease cleavage and plays a multifaced role in the immune response, including types II, V, and VI [18,19]. The simplicity and definite mechanism of class 2 Cas proteins have propelled them to the forefront of biotechnological research [20,21,22]. In CRISPR/Cas nucleic acid detection technology, Cas12a, a member of the Type V Cas proteins, stands out for its straightforward structure and efficacy, rendering it a leading candidate for biosensor-nucleic acid detection [23,24]. Owing to its ease of use, rapid turnaround time, cost-effectiveness, and an unparalleled combination of sensitivity and specificity, in addition to nucleic acid detection, CRISPR/Cas12a-based biosensing has been extended to various non-nucleic acid targets, including small molecules, metal ions, and enzymes, by coupling target recognition with aptamer-mediated or chemically triggered activation strategies [25,26,27]. These advances illustrate the adaptability of Cas12a systems and their potential in broader biosensing applications such as environmental monitoring, food safety assessments, and clinical diagnostics beyond genetic targets [28,29,30,31,32,33,34,35].

Compared with previous reviews, the present review adopts a broader scope, combining mechanistic insights into CRISPR/Cas12a-based biosensors with advances in this detection technology. This review offers the first systematic analysis of its pan-disease diagnostic utility—spanning viral, bacterial, tumor, genetic, and multisystem disorders—discusses the advantages and disadvantages of improving the detection system and applying microfluidic chips, and highlights the innovations and challenges of this technology in clinical application. Our work complements and extends prior reviews by offering a cross-sectional analysis that bridges fundamental mechanisms, engineering innovations, and application outlooks.

## 2. Principles of Cas12a Nucleic Acid Detection Technology for Biosensors

**Figure 1:**
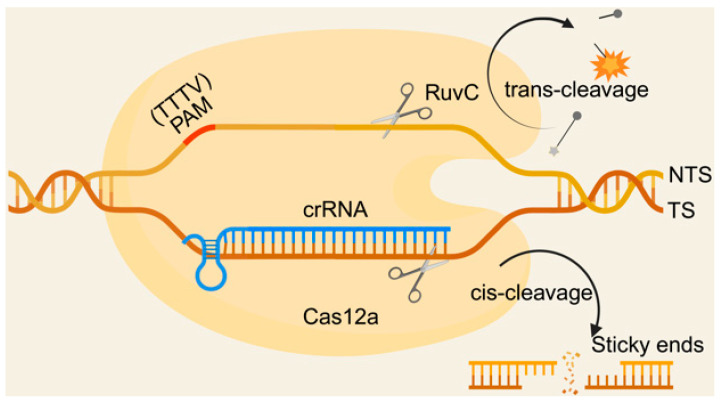
Schematic diagram of the principle of CRISPR/Cas12a-based biosensors.

The Cas12a protein, also known as Cpf1 [16], specifically recognizes dsDNA with a T-rich Protospacer adjacent motif (PAM) upstream of the target sequence and generates a sticky end distal to the PAM site guided by a single CRISPR RNA (crRNA) [36,37]. The crRNA forms an R-loop with the target strand (TS), unmasking the active site of the conserved RuvC nuclease domain within Cas12a and cleaving the non-target strand (NTS), causing the target DNA to unwind and release TS, which is then cleaved by the RuvC domain [38]. This precise cleavage of dsDNA is termed cis-cleavage. Following dsDNA cleavage, the active site of RuvC is released from both the NTS and TS, facilitating the nonspecific cleavage of any surrounding single-stranded DNA (ssDNA) [37,39]. This cleavage of ssDNA is called trans-cleavage (Figure 1).

In CRISPR/Cas12a assays, the specific binding of crRNA to the target dsDNA triggers non-specific ssDNA cleavage activity of the Cas12a protein, which is a key principle for nucleic acid detection. By leveraging this property, ssDNA-based fluorescent probes have been incorporated into the system. The activation of Cas12a induces probe cleavage, separating fluorophores from their respective quenchers at the 5′ and 3′ ends and resulting in a detectable fluorescence signal, which allows for the detection of target DNA through fluorescence intensity measurements [40,41]. Consequently, this technology is particularly suitable for the detection of pathogens and genetic mutations and represents a sensitive and specific diagnostic tool.

## 3. Advances in CRISPR/Cas12a-Based Biosensors for Nucleic Acid Detection in Disease Diagnosis

### 3.1. CRISPR/Cas12a-Based Biosensors for Viral Detection

Doudna et al. revealed that CRISPR/Cas12 targets activated, non-specific ssDNA cleavage and developed the DNA endonuclease-targeted CRISPR trans reporter (DETECTR) for infectious disease detection [42]. They utilized recombinase polymerase amplification (RPA) to amplify the target DNA, which was then detected using CRISPR/Cas12a, demonstrating that DETECTR enables the rapid and specific detection of human papillomavirus (HPV) 16/18 (Table 1).

Severe acute respiratory syndrome coronavirus (SARS-CoV-2) outbreaks have considerably threatened human health and survival. Owing to the high infection rate of SARS-CoV-2, convenient detection is crucial for its control and prevention. CRISPR/Cas12a, when combined with RPA, enables the detection of SARS-CoV-2 using a fluorescent probe in a molecular device [43]. However, this method requires the transfer of reagents between multiple tubes, which is not only operationally complex but also poses a risk of aerosol contamination.

**Figure 2:**
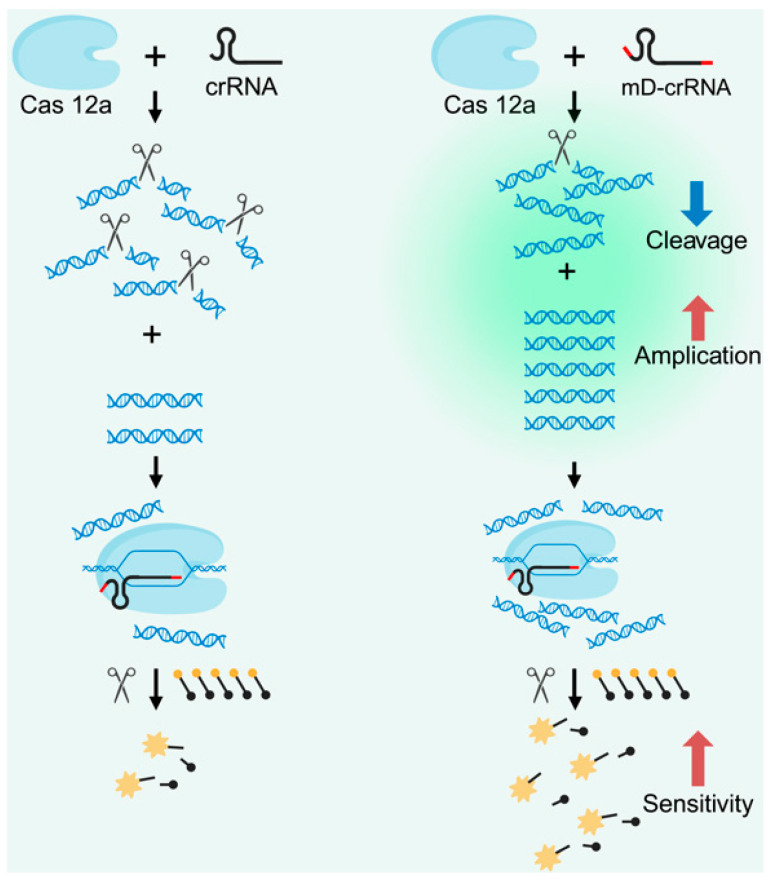
Schematic diagram of CRISPR/Cas12a-based biosensor for the detection of SARS-CoV-2 by modified crRNA.

To avoid this risk, researchers have combined reverse transcription loop-mediated isothermal amplification (RT–LAMP) with Cas12a cleavage in a single reaction system and observed the cutting of fluorescent probes via UV or blue light to detect SARS-CoV-2 [44,45,46]. Other teams have integrated photocontrolled techniques and CRISPR/Cas12a using light irradiation to activate the CRISPR/Cas12a reaction system after RPA amplification is completed in the same tube [47,48]. During the course of the experiment, we found that crRNA was easily degraded by RNases, which considerably affected the accuracy of detection. Our team developed an ssDNA-modified crRNA/Cas12a one-step assay for the detection of SARS-CoV-2, which demonstrated that ssDNA-modified crRNAs increase the sensitivity of the assay and exhibit greater stability than do unmodified crRNAs under RNase treatment [49] (Figure 2).

Except for SARS-CoV-2, other viruses closely related to the quality of life of humans, including African swine fever virus (ASFV) [50,51,52], Mpox virus [53,54], porcine circovirus 3 [55], herpes simplex virus [56], rabies virus [57], rhinovirus (RhV), and human adenovirus (HAdV), can also be proactively and rapidly detected using CRISPR/Cas12 technology [58]. Our team achieved one-step high-sensitivity detection of *Streptococcus agalactiae* (GBS), HPV16, and HPV18 plasmids within 30 min by simply modifying the crRNA [59]. The technique demonstrated 100% accuracy in 5 DNA samples with known HPV genotypes and maintained 100% concordance with qPCR, a commonly used nucleic acid detection method, in 10 GBS clinical samples.

Given that multiple viruses can co-infect the same host, methods based on CRISPR/Cas12a technology for the detection of multiple viruses are becoming increasingly refined. For instance, a microfluidic chip-coupled fluorescent probe and lateral flow assay for simultaneous detection of various SARS-CoV-2 variants have been developed [60,61], achieving the concurrent detection of SARS-CoV-2 and ASFV [62], as well as the simultaneous detection of influenza A (IA), influenza B (IB), respiratory syncytial virus (RSV), and SARS-CoV-2 [63,64]. Beyond conventional in vitro fluorescence assays, recent studies have demonstrated the utility of CRISPR/Cas12a in intracellular imaging and in vivo virus tracing. For example, Li et al. developed an allosteric activator-regulated CRISPR/Cas12a platform that enables biosensing and visualization of both endogenous and exogenous targets in living cells [65]. Similarly, virus-mimicking nanoprobes incorporating Cas12a components have been engineered for ultra-sensitive and accurate imaging of viral infections in vivo, providing new insights into viral pathogenesis and dynamics [66]. These innovations expand the scope of CRISPR/Cas12a beyond traditional biosensing toward real-time, spatially resolved biomedical applications.

CRISPR/Cas12a-based biosensors targeting SARS-CoV-2 and other viruses have optimized substrate amplification, fluorescence techniques, and different visualization media. These methods have faster detection times, lower detection limits, and more efficient workflows than do conventional RT–qPCR assays. The development of CRISPR-based biosensors not only offers a rapid and convenient platform for detecting existing viruses but also provides a robust method for diagnosing infectious diseases from potential viral outbreaks.

### 3.2. CRISPR/Cas12a-Based Biosensors for Detection of Mycoplasma and Bacteria

Concurrently with the development of the DETECTR system, Wang et al. developed the HOLMES (one-HOur Low-cost Multipurpose highly Efficient System), which utilizes the trans-cleavage activity of Cas12a-based biosensor. Initially, the system used PCR for target DNA amplification, followed by target detection based on the cleavage activity of Cas12a [67]. Subsequently, the team replaced PCR with RPA for target DNA amplification, amplifying primers from the conserved region of the Mycoplasma 16s rRNA gene, followed by target detection through the cleavage activity of a Cas12a-based biosensor [68]. This approach integrated RPA and Cas12a into a single detection system that could visually detect Mycoplasma contamination under blue light within 30 min. This technology was validated in 20 clinical samples, demonstrating 100% accuracy.

CRISPR/Cas12a-based biosensors are applicable for the detection of both common bacteria, such as *Mycoplasma pneumoniae* [69,70], Salmonella [71], *Listeria monocytogenes* [72], and *Mycobacterium tuberculosis* [73], as well as rare bacteria, such as *Aspergillus fumigatus* [74] and *Burkholderia pseudomallei* [75]; cleaved using CRISPR/Cas12a after amplified by RPA, these can be detected within 90 min using a lateral flow assay (LFA).

For both common and rare bacteria, the detection of a single bacterium using CRISPR/Cas12a-based biosensors is limited. Similar to the multi-channel assays discussed above, as research progresses, paraffin-isolation RPA and CRISPR/Cas12a-based biosensors can be visualized under LED blue light, allowing for the simultaneous detection of 12 common respiratory pathogens in a single step, including 6 bacteria and 6 viruses (e.g., *Streptococcus pneumoniae*, *Staphylococcus aureus*, *Pseudomonas aeruginosa*, *Haemophilus influenzae*, *Klebsiella pneumoniae*, and *Acinetobacter baumannii*) with a detection sensitivity of 2.5 copies/μL [58].

These applications broaden the utility of CRISPR/Cas12a-based biosensors in bacterial and viral diagnostics and offer new possibilities for the detection of infectious diseases (Table 2).

### 3.3. CRISPR/Cas12a-Based Biosensors for Parasite Detection

In recent years, the range of parasite detection using CRISPR/Cas12a-based biosensors has expanded, and advancements in the visualization of inspection results have been particularly noticeable. Researchers combined RPA with CRISPR/Cas12a-based biosensors and employed lateral flow band readouts to detect the pathogen *Theileria parva* in bovine blood samples to diagnose East Coast fever (ECF) [76]. This technique was capable of detecting eight strains of T. parva, with detection limits consistent with those of existing techniques for East Coast fever detection. The entire process, from sampling to result interpretation, can be completed in <2 h. Researchers applied this technique to successfully identify infected individuals using 16 samples from cattle. This is the first known detection method that utilizes the CRISPR/Cas12a system to diagnose East Coast fever, facilitating the detection of *T. parva* (Table 3).

The use of LFAs to observe detection results can be challenging in terms of stability and timeliness. However, there are many other ways to visualize test results that not only compensate for the lack of test strips but also increase the flexibility of the inspection process. Researchers have employed RPA in conjunction with CRISPR/Cas12a-based biosensors and combined it with fluorescence reporter systems to detect Neospora [77]. The entire inspection process required 90 min, and the test results were visualized on LFA or under UV and blue light. Placental tissue and canine fecal samples tested using this technique showed complete agreement with nested PCR results. Similarly, RPA combined with the CRISPR/Cas12a-based biosensors proved effective in detecting toxoplasmosis in dogs and cats [78]. This technique leverages digital visualization to avoid false negatives associated with weakly positive samples when using LFA.

Previous studies have demonstrated that CRISPR/Cas12a-based biosensors can detect infectious diseases caused by various parasites such as zoonotic nematode Anisakis [79], bovine *Anaplasma marginale* [80], *Opisthorchis viverrine* [81], *Clonorchis sinensis* [82], *Heterodera avenae* and *Heterodera filipjevi* [83], Plasmodium spp. parasites [84], *Schistosoma haematobium* [85], and *Leishmania species* [86]. Researchers have often focused on detecting parasite species rather than modifying the CRISPR/Cas12a-based biosensors themselves. This indicates that the application of these biosensors for parasite detection can be continuously optimized. Using CRISPR/Cas12a-based biosensors to detect parasitic infections not only reduces the economic losses of livestock farming at a lower cost but also enables early prevention and treatment of zoonotic diseases.

### 3.4. CRISPR/Cas12a-Based Biosensors for Tumor Detection

Research on the use of the CRISPR/Cas12a-based biosensors for tumor detection can be categorized into three main areas—detection of gene mutations, identification of tumor biomarkers, and detection of high-risk factors for tumorigenesis (Table 4).

To detect gene mutation sites, Xu et al. designed a disk-shaped microfluidic chip that was specifically cleaved by the Mscl restriction enzyme to facilitate recombinase-assisted isothermal amplification (RAA) for the specific amplification of low-abundance mutant genes in epidermal growth factor receptor exon 21 (EGFR L858R). The detection of these low-abundance mutant genes with CRISPR/Cas12a-based biosensors can further aid in the diagnosis of non-small cell lung cancer (NSCLC) [87]. The device not only extracts DNA but also analyzes four blood samples simultaneously, with the test results visualized under blue light within 1 h. This technique was validated using 10 clinical samples. To detect gene mutation sites, we combined RPA with the CRISPR/Cas12a-based biosensors to diagnose chromosome-negative myeloproliferative neoplasms (MPNs) in Philadelphia by detecting JAK2 V617F mutations using LFA [88]. The method requires a minimum of 0.01 ng/μL of gDNA and achieves a maximum sensitivity of 0.01%, which is two orders of magnitude higher than what is required for clinical applications. The entire process is completed within 1.5 h. In 14 clinical samples, this method showed 100% agreement with NGS.

To detect tumor biomarkers, Guan et al. integrated the CRISPR/Cas12a-based biosensors with an aptamer chemiluminescence assay (CACBA) to quantify and determine the relative abundance of tumor-associated protein-positive exosomes, which were used to distinguish breast cancer patients from healthy individuals [89]. In addition, CRISPR/Cas12a-based biosensors have shown promise for the detection of lung cancer biomarkers; researchers developed a fan- and dumbbell-shaped probe as a template for crRNA enrichment through rolling circle transcription (RCT) to simultaneously detect human 8-hydroxyguanine DNA glycosidase (hOGG1) and flap endonuclease 1 (FEN1), both of which are closely associated with lung cancer [90].

CRISPR/Cas12a-based biosensors can detect high-risk carcinogens, such as mycotoxins, and small-molecule contaminants that pose a threat to human health, including heavy metals [91]. In this approach, researchers utilize the preferential selective binding of an active DNA probe to a microplate to influence the activation of Cas12a and further decrease the cleavage of nonspecific fluorescent probes. This method leverages the signal amplification capability of CRISPR/Cas12a-based biosensors, achieving detection limits of 31 pM for aflatoxin B1 and 3.9 nM for Cd ions. Li et al. examined cytotoxin-associated gene A (CagA) and vacuolar cytotoxin A (VacA), which contribute to the pathogenicity of *Helicobacter pylori*, to aid in the prevention and monitoring of cancerous lesions [92]. Using Triton X-100, the team lysed clinical samples within 2 min, combined them with loop-mediated isothermal amplification (LAMP), and employed optimized buffers to enhance Amor sensitivity. The developed buffer allowed Cas12a to achieve picomolar sensitivity (171 pM) without requiring target pre-amplification, resulting in a 16-fold increase in trans-cleavage activity.

In addition to the aforementioned tumors, CRISPR/Cas12a-based biosensors have shown significant potential for the detection of various other cancers, including nasopharyngeal carcinoma [93], esophageal cancer [94], bladder cancer [95], hepatocellular carcinoma [96], and prostate cancer [97]. The profound impact of cancer on individual health and the associated family burden are well documented. The application of CRISPR/Cas12a-based biosensors in tumor diagnosis not only has the potential to reduce overtreatment but also offers valuable support for subsequent treatment planning and prognosis evaluation.

### 3.5. CRISPR/Cas12a-Based Biosensors for Genetic Disease Detection

CRISPR/Cas12a-based biosensors are also beneficial for the auxiliary diagnosis of certain genetic diseases. Using the CRISPR/Cas12a detection platform combined with ERA target DNA amplification, the three major variants of Leber’s hereditary optic neuropathy (LHON) can be detected within 30 min using only a drop of blood, with the sensitivity significantly surpassing that of Sanger sequencing [98]. This method demonstrated accuracy equivalent to that of Sanger sequencing and NGS in 182 clinical samples. However, from the perspective of detection design, this technology is limited by the PAM sequence, which requires the presence of a special PAM sequence on the target DNA to detect a locus in the sequence. Moreover, from the perspective of detection, the technology is completed in two steps, which may interfere with the detection results owing to aerosol contamination.

**Figure 3:**
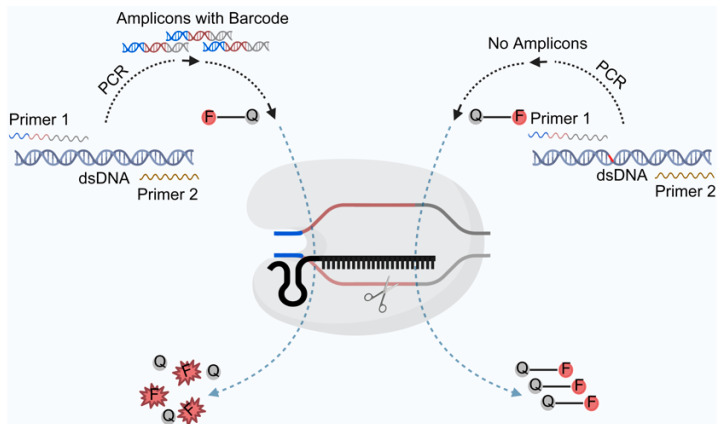
Schematic diagram of a CRISPR/Cas12a-based biosensor for the detection of β-thalassemia without PAM or crRNA limitations.

To address these problems, our team developed a DNA detection technique using CRISPR/Cas12a-based biosensors, which integrates crRNA into a reporter probe for the detection of genetic variants [41]. We designed a probe hybridization strategy that identifies the target DNA without the limitations of PAM sequences (Figure 3). Multiple mutation sites in β-thalassemia (Eastern Mediterranean type) can be detected simultaneously in a single reaction, with quantitative detection capabilities that can even distinguish two-fold differences in copy numbers. After optimizing the reaction conditions, this method can differentiate patients with spinal muscular atrophy (SMA), carriers, and healthy individuals in a single tube, providing valuable guidance for population screening of genetic diseases.

**Figure 4:**
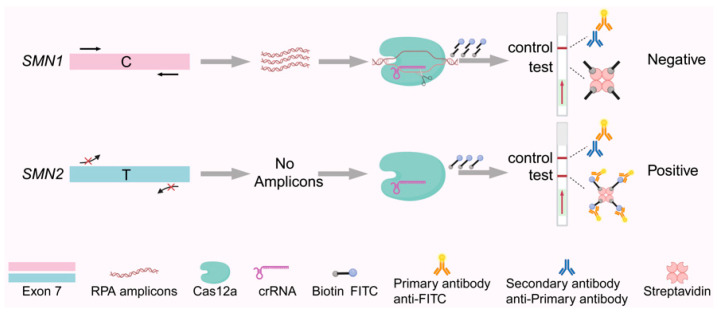
Schematic diagram of a CRISPR/Cas12a-based biosensor for the detection of spinal muscular atrophy (SMA).

Subsequently, our team combined RPA with LFA to develop an SMA detection technology that provides visual results within 1.5 h [99]. By artificially introducing a mismatch adjacent to the mutation site in crRNA, we achieved a 7.41-fold difference in fluorescence between patients with SMA and healthy individuals (Figure 4). This CRISPR/Cas12a-based biosensor, validated in 168 clinical samples, demonstrated 100% sensitivity and specificity with a detection limit as low as 526 aM. This biosensor has also been validated in clinical samples from patients with Duchenne muscular dystrophy (DMD).

To date, the application of CRISPR/Cas12a-based biosensors in genetic diseases has been studied far less extensively than its use in detecting pathogenic infections, tumors, and other higher-incidence diseases. Nevertheless, current research indicates that CRISPR/Cas12a-based biosensors have significant potential for advancing the field of genetic disease detection. These biosensors are expected to enable widespread genetic disease screening, ultimately contributing to reduced morbidity and mortality rates (Table 4).

### 3.6. CRISPR/Cas12a-Based Biosensors for Multisystem Disease Detection

CRISPR/Cas12a-based biosensors are useful for detecting variants associated with multisystem diseases such as miRNA-related diseases, aberrant DNA methylation-related diseases, and nicotinamide adenine dinucleotide (NAD^+^)-related diseases.

MiRNAs (microRNAs) are believed to be closely linked to cell differentiation, biological evolution, and disease progression [103,104]. Among non-infectious diseases, miRNA-1290 is notably overexpressed in patients with pancreatic cancer. Similarly, patients with breast cancer show elevated levels of miRNA-141, miRNA-155, and miRNA-21 [105], whereas miRNA-141 and miRNA-375 have been associated with prostate disease [106]. These miRNAs not only represent new therapeutic targets [107,108] but also serve as biomarkers for disease diagnosis [109]. Using CRISPR/Cas12a-based biosensing, researchers have developed a DNA hairpin containing a PAM sequence that binds to two classes of miRNAs [100]. When both the miRNAs are present, they bind to the DNA hairpin, which is processed by Exo III. This digestion deactivates the trans-cleavage activity of Cas12a-based biosensors, preserving the integrity of the T-DNA of the silver nanocluster template strand and allowing for the synthesis of DNA-AgNCs with a strong fluorescent signal. This method has a detection limit of 84 fmol/L and has been validated in human serum samples and cell lysates. This CRISPR/Cas12a-based biosensor introduces a novel approach for simultaneous quantitative detection of multiple nucleic acids.

Aberrant DNA methylation [110,111,112] is associated with many diseases. Accurate analysis of DNA methylation levels is crucial for monitoring disease onset and progression, as well as for developing tailored treatment plans, and they play an essential role in clinical diagnosis and therapy. CRISPR/Cas12a-based biosensing has been utilized to achieve the relative quantification of DNA methylation levels within 6 h, which is a significant improvement over traditional bisulfite treatment [101]. This method incorporates PAM sequences into the primer design, enabling amplification at any site by PCR, with results visualized using fluorescent agents or LFA to detect DNA methylation.

NAD^+^ is intricately linked to metabolic diseases, and its quantitative detection provides insights into the progression of these conditions, thereby reflecting the overall state of an organism. One study developed a method involving one-step acetylation of CRISPR/Cas12a-based biosensing using V-A anti-CRISPR protein 5 (AcrVA5), which is essential for CobB-mediated deacetylation and reactivation of Cas12a. This approach allows for quantification in a single step, combined with a fluorescent probe, and can be completed within 30 min at 37 °C [102]. The method was validated using biospecimens, and the results were consistent with those obtained using liquid chromatography/electrospray ionization tandem mass spectrometry (MS) and MTT colorimetry (Table 4).

Research into the application of CRISPR/Cas12a-based biosensors for multisystem disease markers suggests that in today‘s era of overlapping disease pathways, this technology has the potential to quantitatively detect common factors driving multiple diseases. This capability can support the identification of therapeutic targets and advance the realization of precision medicine.

## 4. Innovations and Challenges of CRISPR/Cas12a-Based Biosensors in Clinical Research

**Figure 5:**
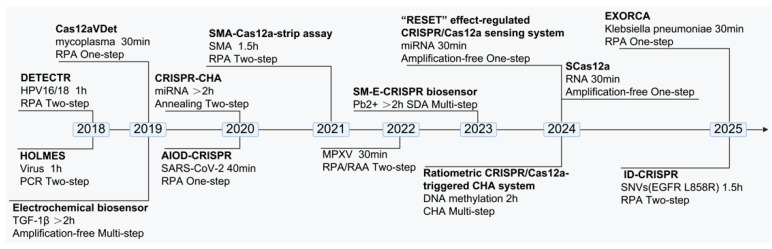
Timeline of technological advancements in CRISPR/Cas12a-based nucleic acid detection.

Since Jennifer et al. reported Cas12a-based biosensors in 2018 [42], researchers have incorporated amplification methods to shorten the detection time and eliminate the need for precision instruments [70,72,113,114,115], developed various detection platforms [87,116,117,118], targeted dsDNA amplification, and gradually integrated Cas12a-based biosensors into single reaction systems [114,119]. Techniques such as lateral flow dipsticks and UV/blue light visualization can be used to display results [58,70,81,119]. These approaches not only help prevent aerosol contamination but also aid in streamlining the procedure and enhancing detection accuracy (Figure 5). In addition, some studies have developed Cas12a-based biosensors to eliminate the need for nucleic acid amplification [51,93], thereby reducing the number of steps required for testing. Further research has focused on engineering the Cas12a protein or crRNA to improve detection efficiency by modifying their biological structures [49,102,120,121]. As exploration of Cas12a-based biosensors continues, their applications have expanded from DNA to RNA detection, with some studies demonstrating that Cas12a-based biosensors can directly detect RNA [122].

Studies on assay components have reported that the addition of Mg^2+^ and Mn^2+^ to the system can enhance both sensitivity and specificity [120,123]. Research on reporter probes indicates that within a certain range, longer T- and A-rich fluorescent probes generate stronger fluorescence signals and more rapid responses. Notably, the efficiency of a fluorescent probe composed of 10 T bases is the most pronounced [120]. These findings have significantly improved the efficiency of CRISPR/Cas12a-based biosensors, enabling the detection of trace amounts of various diseases.

To study the reaction process, researchers have developed various microfluidic chips and microwell plates aimed at amplifying detection signals while simplifying the reaction steps. These microfluidic chips contribute to intuitive operation, reduce aerosol contamination, and enhance detection sensitivity to some extent. However, these methods are often complex and involve multiple sequential processes on a single sensor, and some require intricate probe hybridization to analyze multiple targets simultaneously. Consequently, achieving low cost and accuracy typical of simpler single-tube reactions remains challenging.

Despite its potential, CRISPR/Cas12a-based biosensors face several challenges in clinical applications. (1) PAM sequence limitations: The dependence of Cas12a-based biosensors on PAM sequences restricts the selection of target DNA and the design of CRISPR RNAs (crRNAs). When the PAM sequence is absent from the target DNA, it must be introduced to the PAM sequence to facilitate Cas12a-based biosensor identification. Some studies have attempted to overcome this limitation using dual crRNAs, which have been reported to offer higher sensitivity than that of single crRNAs [124]. However, the efficacy of this approach across diverse diseases remains uncertain because of variability in crRNA tolerance for target DNA recognition. (2) Lack of absolute quantification: Current research primarily focuses on detecting the presence or absence of target DNA, with some studies achieving relative quantification through the observation of fluorescence efficiency. Absolute quantification of target DNA, which is crucial for monitoring disease progression and guiding treatment regimens, is not yet feasible using most CRISPR/Cas12a-based biosensors. (3) Limited disease applicability: CRISPR/Cas12a-based biosensors are currently effective only for detecting diseases with known genetic sequences. This limitation reduces the scope of its application and prevents its use for a broad range of diseases. (4) Translation to clinical practice: Most research on CRISPR/Cas12a-based biosensors remains theoretical and has not been extensively validated in large-scale clinical settings. Although some studies have provided clinical validation, the practical implementation of the biosensors and challenges encountered in actual testing have not yet been fully addressed. Despite advances in proof-of-concept studies and preliminary clinical validation, several obstacles continue to hinder the practical application of CRISPR/Cas12a-based biosensors in real-world settings. These include limitations in conducting high-throughput testing, the absence of compact and integrated devices for point-of-care analysis, and difficulties in adapting laboratory protocols for clinical workflows [33,35]. Moreover, translating these technologies into user-friendly and reliable diagnostic platforms remains a major challenge. Future efforts should prioritize multi-center clinical trials, the development of portable and automated detection systems, and the establishment of clear regulatory and manufacturing standards to support broader clinical adoption.

These challenges represent significant obstacles to leveraging CRISPR/Cas12a-based biosensors for early disease screening and maximizing its public health benefits.

## 5. Summary

As a cutting-edge nucleic acid detection technology, CRISPR/Cas12a-based biosensors have shown substantial promise for diagnosing and researching a wide array of conditions, including infectious diseases (such as those caused by viruses, bacteria, and parasites), tumors, genetic disorders, and other non-infectious diseases. Overcoming current limitations—such as the strict dependence on PAM sequences, inability to achieve absolute quantification, and narrow scope of clinical applications—will be critical to advancing CRISPR/Cas12a-based biosensors in nucleic acid detection. Future research should prioritize the rational design of crRNAs, the development of one-pot reaction systems, and the establishment of high-throughput detection platforms. Continued innovation will likely enable point-of-care diagnostics and disease screening in remote areas, thereby substantially contributing to global health.

## Figures and Tables

**Table 1:** Application of CRISPR/Cas12a-based biosensors in viruses.

Virus	Amplification	Visualization	Sensitivity	Detection Time	One/Two-Step	Reference
HPV16/18	RPA	Fluorescence	/	1 h	Two-step	[42]
SARS-CoV-2	RPA	Fluorescence	0.4 copies/μL	50 min	Two-step	[43]
SARS-CoV-2	LAMP	Fluorescence (portable device)	10 copies/μL	40 min	One-step (physically separated)	[44]
SARS-CoV-2	RT-LAMP	Fluorescence (naked eye/blue light)	5 copies/μL	45 min	One-step (physically separated)	[45]
SARS-CoV-2	RT-LAMP	Fluorescence (naked eye)	30 copies/μL	40 min	One-step (physically separated)	[46]
SARS-CoV-2	RT-RPA	Fluorescence	10 copies/μL	30 min	One-step (optochemical control)	[47]
SARS-CoV-2 variants	RPA	Fluorescence	30 copies/μL	1 h	One-step (optochemical control)	[48]
SARS-CoV-2	RT-RPA	Fluorescence (UV)	5 aM	50 min	One-step	[49]
ASFV	RPA	Fluorescence (blue light)/LFD	6.8 copies/μL	1 h	Two-step	[50]
ASFV	Amplification-free	magnetic-SERS nanoprobe	10 fM	2 h	Multi-step	[51]
ASFV	LAMP	Fluorescence	1 copies/μL	50 min	One-step (physically separated)	[52]
MPXV	PCSDA	Fluorescence	2.8 × 10^−4^ ng/μL	100 min	Two-step	[53]
MPXV	RPA	Fluorescence/LFA	1 copies/μL	45 min	Two-step	[54]
PCV3	ERA	Fluorescence	1.16 copies/μL	1 h	Two-step	[55]
HSV-1	Amplification-free	Electrochemical signal	3 aM	6.5 h	Multi-step	[56]
PRV	MIRA	Fluorescence (blue light)	1.65 × 10^4^ copies/μL	25 min	Two-step	[57]
12 respiratory pathogens *	RPA	Fluorescence	2.5 × 10^0^ copies/μL	90 min	One-step (physically separated)	[58]
GBS, HPV16/18	RPA	Fluorescence	16.6 aM	30 min	One-step	[59]
H_1_N_1_, H_3_N_2_, IVB, HRSV, SARS-CoV-2 variants	RPA	Fluorescence	0.1 copies/μL	1 h	One-step (microfluidic chip)	[60]
SARS-CoV-2 variants	RT-RPA	Fluorescence (UV/blue light)	0.01 copies/μL	1 h	Two-step	[61]
SARS-CoV-2/ASFV	RPA/RT-RPA	Fluorescence (portable device)	8 copies/μL	1.5 h	Two-step	[62]
IA, IB, RSV, SARS-CoV-2	RPA	Fluorescence (portable device/naked eye)	0.8 copies/μL	1.5 h	One-step (microfluidic chip)	[63]
SARS-CoV-2 variant, IA, IB, RSV, IC	RT-RPA	Fluorescence (portable device)	1 copies/μL	40 min	One-step (physically separated)	[64]

“/” indicates not applicable. * 12 respiratory pathogens, including *Streptococcus pneumoniae*, *Haemophilus influenzae*, *Klebsiella pneumonia*, *Pseudomonas aeruginosa*, *Acinetobacter baumannii*, *Staphylococcus aureus*, SARS-CoV-2, Influenza A virus, Influenza B virus, Respiratory Syncytial Virus, rhinovirus, and human adenovirus.

**Table 2:** Application of CRISPR/Cas12a-based biosensors in mycoplasma and bacterium.

Pathogens	Amplification	Visualization	Sensitivity	Detection Time	One/Two-Step	Reference
mycoplasma	RPA	Fluorescence (Blue Light)	10 aM	30 min	One-step (physically separated)	[68]
*M. pneumoniae*	ERA	Fluorescence/LFA	1 copies/μL	30 min	Two-step	[69]
*M. pneumoniae*	MCDA	Fluorescence	50 fg	50 min	Two-step	[70]
*Salmonella enterica*	LAMP	Fluorescence	20 CFU	<1 h	Two-step	[71]
*Listeria monocytogenes*	RAA	Fluorescence	0.68 aM	40 min	Two-step	[72]
*Mycobacterium tuberculosis*	LAMP	Fluorescence/LFA	50 fg	1 h	Two-step	[73]
*Aspergillus fumigatus*	RPA	Fluorescence/LFA	10^2^ copies/µL	40 min	Two-step	[74]
*Burkholderia pseudomallei*	RPA	LFA	50 CFU/mL	90 min	Two-step	[75]

**Table 3:** Application of CRISPR/Cas12a-based biosensors in parasites.

Parasites	Amplification	Visualization	Sensitivity	Detection Time	One/Two-Step	Reference
*Theileria parva*	RPA	Fluorescence/LFA	1 infected lymphocyte/3 μL	80 min	Two-step	[76]
*Neospora caninum*	RPA	Fluorescence/LFA	1 parasites/mL	90 min	Two-step	[77]
*Toxoplasma gondii*	RPA	Fluorescence/LFA	31 copies/μL	55 min	Two-step	[78]
Anisakis	RPA	Fluorescence (naked eye/portable device)	31.6 copies/μL	80 min	One-step (physically separated)	[79]
*Anaplasma marginale*	RPA	Fluorescence/LFA	4 copies/μL	<1 h	Two-step	[80]
*Opisthorchis viverrini*	RPA	Fluorescence (UV)	1 ng	<1 h	Two-step	[81]
*Clonorchis sinensis*	RPA	Fluorescence/LFA	1 copies/μL	<1 h	One-step (physically separated)	[82]
*Heterodera avenae/Heterodera filipjevi*	RPA	Fluorescence/LFA	10^−4^ J2	<1 h	Two-step	[83]
*Plasmodium* spp.	RPA	Fluorescence/LFA	3.11–7.27 parasites/μL	<1 h	Two-step	[84]
*Schistosoma haematobium*	RPA	Fluorescence/LFA	2 eggs	70 min	One-step	[85]
*Leishmania*	PCR	Fluorescence	1–42 parasites/10^6^ human cells	<1 h	Two-step	[86]

**Table 4:** Application of CRISPR/Cas12a-based biosensors in tumors, genetic diseases, and multiple-system diseases.

Diseases/Biomarkers	Amplification	Visualization	Sensitivity	Detection Time	One/Two-Step	Reference
NSCLC	RAA	Fluorescence (blue light)	10 copies/μL	<1 h	Two-step (microfluidic chip)	[87]
MPN	RPA	Fluorescence/LFA	3 copies/μL	1.5 h	Two-step	[88]
Breast cancer	Amplification-free	Fluorescence	1.45 × 10^2^/3.73 × 10^2^ particles/μL	1 h	Multi-step	[89]
CCBMH	Amplification-free	Fluorescence	AFB1: 31 pM Cd^2+^: 3.9 nM	<2 h	Multi-step	[90]
Osteosarcoma	Amplification-free	Fluorescence	3 HeLa cells	70 min	Multi-step	[91]
Stomach cancer	LAMP	Fluorescence/LFA	43 aM	<1 h	One-step	[92]
NPC	Amplification-free	Fluorescence (inverted microscope)	5 copies/μL	<2 h	One-step (microfluidic chip)	[93]
Esophageal cancer	Amplification-free	Fluorescence	1.26 fM	<3 h	Multi-step	[94]
BCa	RT-RAA	Fluorescence	0.1 copies/μL	30 min	Twp-step	[95]
HCC	TTSD	Colorimetric assays	0.5 pM	<2 h	Multi-step	[96]
Prostate cancer	Amplification-free	Colorimetric assays (portable device)	f-PSA: 0.04 ng/mL t-PSA: 0.06 ng/mL	<1 h	Multi-step	[97]
LHON	ERA	Fluorescence (blue light)	/	30 min	Two-step	[98]
SMA	PCR/RPA	Fluorescence/LFA	526 aM	1.5 h	Two-step	[99]
miRNA-155/miRNA-141	Amplification-free	Fluorescence	84 fmol/L	<2 h	Multi-step	[100]
methylated DNA	PCR	Fluorescence/LFA	/	6 h	Multi-step	[101]
NAD^+^	Amplification-free	Fluorescence	22.5 nM	30 min	One-step	[102]

“/” indicates not applicable.

## Data Availability

Not applicable.

## Abbreviations

The following abbreviations are used in this manuscript:

PCR–RFLP	Polymerase chain reaction–restriction fragment length polymorphism
MLPA	Multiplex ligation-dependent probe amplification
DHPLC	Denaturing high-performance liquid chromatography
qPCR	Quantitative real-time polymerase chain reaction
dPCR	Digital polymerase chain reaction
NGS	Next-generation sequencing
CRISPR	Clustered regularly interspaced short palindromic repeats
Cas	CRISPR-associated
PAM	Protospacer adjacent motif
crRNA	CRISPR RNA
TS	Target strand
NTS	Non-target strand
ssDNA	Single-stranded DNA
RPA	Recombinase polymerase amplification
HPV	Human papillomavirus
SARS-CoV-2	Severe acute respiratory syndrome coronavirus
DETECTR	DNA endonuclease-targeted CRISPR trans reporter
RT-LAMP	Reverse transcription loop-mediated isothermal amplification
ASFV	African swine fever virus
MPXV	Mpox Virus
RhV	Rhinovirus
HAdV	Human adenovirus
GBS	Streptococcus agalactiae
IA	Influenza A
IB	Influenza B
RSV	Respiratory syncytial virus
RT–qPCR	Reverse transcription–polymerase chain reaction
HOLMES	one-HOur Low-cost Multipurpose highly Efficient System
ECF	East Coast fever
RAA	Recombinase-assisted isothermal amplification
NSCLC	Non-small cell lung cancer
MPNs	Myeloproliferative neoplasms
CACBA	Aptamer chemiluminescence assay
RCT	Rolling circle transcription
hOGG1	Human 8-hydroxyguanine DNA glycosidase
FEN1	Flap endonuclease 1
CagA	Cytotoxin-associated gene A
VacA	Vacuolar cytotoxin A
LAMP	Loop-mediated isothermal amplification
LHON	Leber’s hereditary optic neuropathy
SMA	Spinal muscular atrophy
DMD	Duchenne muscular dystrophy
NAD^+^	Nicotinamide adenine dinucleotide
microRNAs	miRNAs
LFA	Lateral flow assay
AcrVA5	V-A anti-CRISPR protein 5
MS	Mass spectrometry
PCSDA	PLA-induced Cascade strand displacement and amplification
MCDA	Multiple cross-displacement amplification
J2	Single second-stage juvenile
TTSD	Toehold-triggered strand displacement
